# Solid-phase capture and profiling of open chromatin by spatial ATAC

**DOI:** 10.1038/s41587-022-01603-9

**Published:** 2023-01-05

**Authors:** Enric Llorens-Bobadilla, Margherita Zamboni, Maja Marklund, Nayanika Bhalla, Xinsong Chen, Johan Hartman, Jonas Frisén, Patrik L. Ståhl

**Affiliations:** 1 Department of Cell and Molecular Biology, Karolinska Institute, Stockholm, Sweden; 2 SciLifeLab, Department of Gene Technology, KTH Royal Institute of Technology, Stockholm, Sweden; 3 Department of Oncology-Pathology, Karolinska Institute, Stockholm, Sweden; 4 Department of Clinical Pathology and Cancer Diagnostics, Karolinska University Hospital, Stockholm, Sweden

**Keywords:** Genomic analysis, Systems biology, Developmental biology

## Abstract

Current methods for epigenomic profiling are limited in their ability to obtain genome-wide information with spatial resolution. We introduce spatial ATAC, a method that integrates transposase-accessible chromatin profiling in tissue sections with barcoded solid-phase capture to perform spatially resolved epigenomics. We show that spatial ATAC enables the discovery of the regulatory programs underlying spatial gene expression during mouse organogenesis, lineage differentiation and in human pathology.

## Abstract (web summary)

Chromatin accessibility profiles are spatially resolved in tissue sections.

## Main

In multicellular organisms, cells progressively acquire specialized gene expression programs according to their position within a tissue^1^. Cell type-specific gene expression patterns result in part from the interaction between the transcriptional machinery and regulatory elements in the chromatin^2,3^, a process dysregulated in disease^4,5^. Several methods have been developed to integrate gene expression and chromatin accessibility measurements in single cells^6–8^. Single-cell methods typically require tissue dissociation, and a wealth of spatial profiling methods has recently been developed to overcome this limitation, particularly at the transcriptome level^9^. However, we remain limited in our ability to interrogate chromatin accessibility with spatial resolution at a comparable scale because current spatial chromatin profiling approaches require custom microfluidics or microbiopsies^10,11^.

**Fig. 1:**
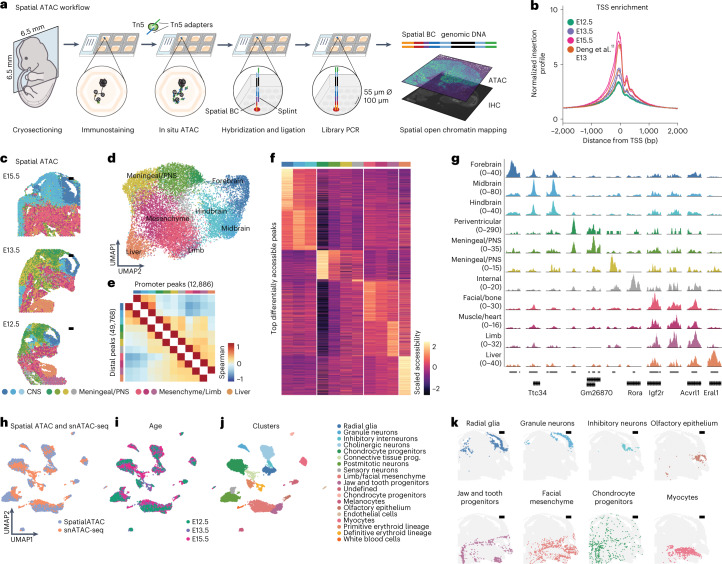
Workflow and spatial mapping of chromatin accessibility in mouse embryos. **a**, Schematic workflow of spatial ATAC. Transposition with Tn5 is performed on immunostained tissue cryosections immobilized on a barcoded slide (55 µm spot diameter; 100 µm interspot distance). Transposed fragments are surface-captured using a splint oligonucleotide, which is ligated and extended to allow the generation of a spatially barcoded DNA library. **b**, Enrichment of ATAC-seq fragments around the TSS in spatial ATAC performed on mouse embryos (E12.5, E13.5, E15.5) in comparison with spatial ATAC-seq E13 data from ref. 11. **c**, Clustering of spatial ATAC open chromatin fragments projected on their spatial location. **d**, UMAP of all spots from mouse embryo sections colored by cluster as in **c**. **e**, Cluster-wise correlation of the accessibility of the top 25% variable promoter (+1,000, −100 bp around the TSS) and distal peaks. **f**, Heatmap showing scaled accessibility of the top differentially accessible peaks per cluster. **g**, Genome tracks showing normalized spatial ATAC-seq fragment density for peaks showing cluster-specific accessibility. Cluster colors are consistent from **c**–**g**. **h**–**j**, UMAP showing the integration of spatial ATAC with snATAC-seq profiles from the same developmental stages colored by technology (**h**), developmental age (**i**) or clustering (**j**). **k**, Individual clusters from **j** projected onto their original spatial location in an E15.5 spatial ATAC section. Scale bars, 500 µm.

We developed spatial ATAC to perform spatially resolved chromatin accessibility profiling in tissue sections. Spatial ATAC combines the assay for transposase-accessible chromatin and sequencing (ATAC-seq^12^) with tagmented DNA capture on a solid surface containing barcoded oligonucleotides, using an experimental platform analogous to our previous spatial transcriptomics approach^13^. First, we immobilize fresh frozen tissue sections onto barcoded slides and crosslink them to preserve chromatin structure during immunostaining. Immunostained sections are then imaged to register tissue coordinates and protein expression data. In the next step, Tn5 transposition is performed directly in permeabilized sections to tagment open chromatin. With the help of a chimeric splint oligonucleotide, DNA tagments are hybridized to spatially barcoded surface oligonucleotides during gentle tissue digestion. Ligation to the splint and subsequent polymerase gap fill and extension generate open chromatin fragments carrying a spatial barcode and PCR handles that are used for generating a sequencing library (Fig. 1a).

**Extended Data Fig. 1:**
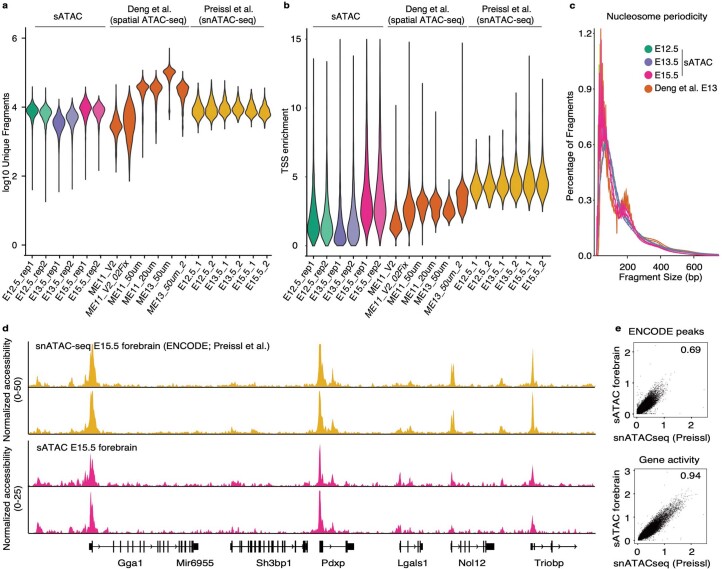
Quality control metrics of spatial ATAC. **a-b**. Violin plot showing unique fragments per spot and TSS enrichment in embryo sections processed using spatial ATAC, spatial ATAC-seq from^10^ and single-nucleus ATAC-seq from^14^. **c**. Fragment size histogram colored by technology and replicate as in Fig. 1b. **d**. Genome ATAC-seq coverage tracks from ENCODE E15.5 forebrain snATAC-seq replicates and aggregate signal from forebrain spots from two spatial ATAC E15.5 sections. **e**. Scatterplot showing the correlation (Spearman’s rho) between log-normalized values for peak (top) and gene (bottom) accessibility for snATAC-seq and spatial ATAC mouse developmental forebrain datasets.

**Extended Data Fig. 2:**
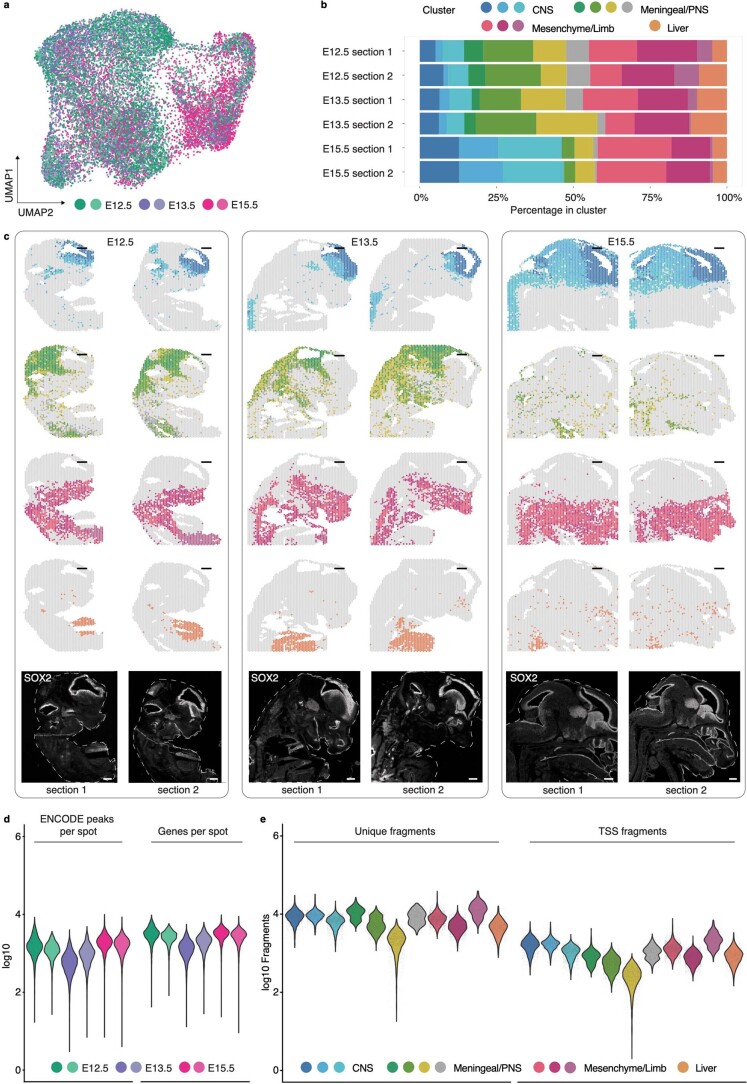
spatial ATAC in mouse embryos. **a**. UMAP embedding corresponding to Fig. 1d colored by embryonic age and section replicate. **b**. Cluster proportions across embryo sections. **c**. Cluster families as in Fig. 1c for all sections analyzed (n = 6, 2 per embryonic stage). SOX2 immunostaining for the respective section at the bottom. Scale bars are 500 µm. **d**. Number of ENCODE peaks and genes detected per spot across sections. **e**. Numbers of unique fragments and TSS fragments across clusters. Each dot corresponds to a tissue spot.

**Extended Data Fig. 3:**
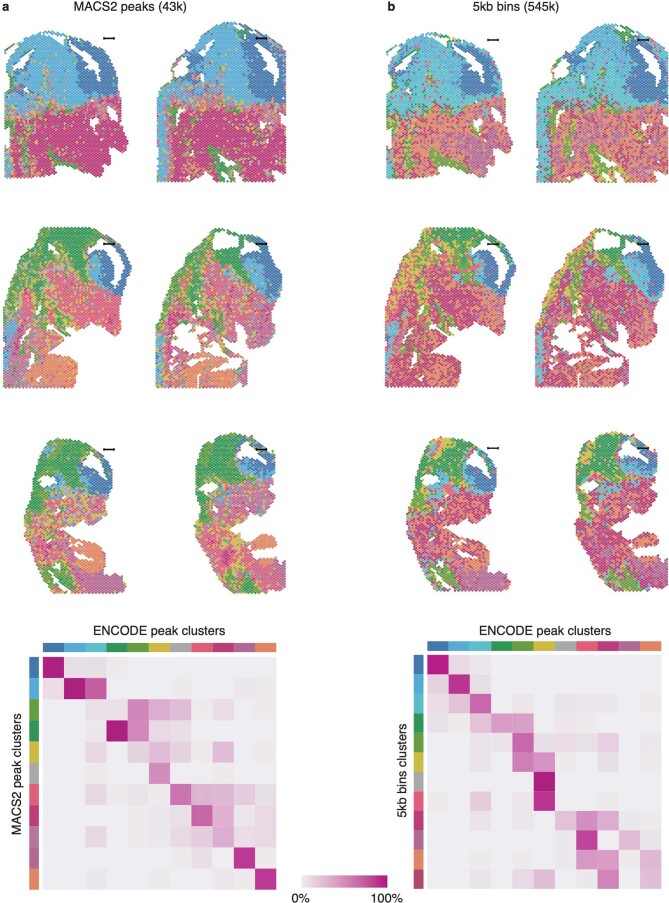
spatial ATAC clustering robustness. **a**. Spatial ATAC clustering on E12.5, E13.5 and E15.5 replicate sections using peaks called by MACS2. Below, correspondence between cluster identities based on ENCODE peaks (Fig. 1c–g) or on MACS2 peaks. **b**. Same as in a, but using fragment counts in 5 kb genomic bins.

**Extended Data Fig. 4:**
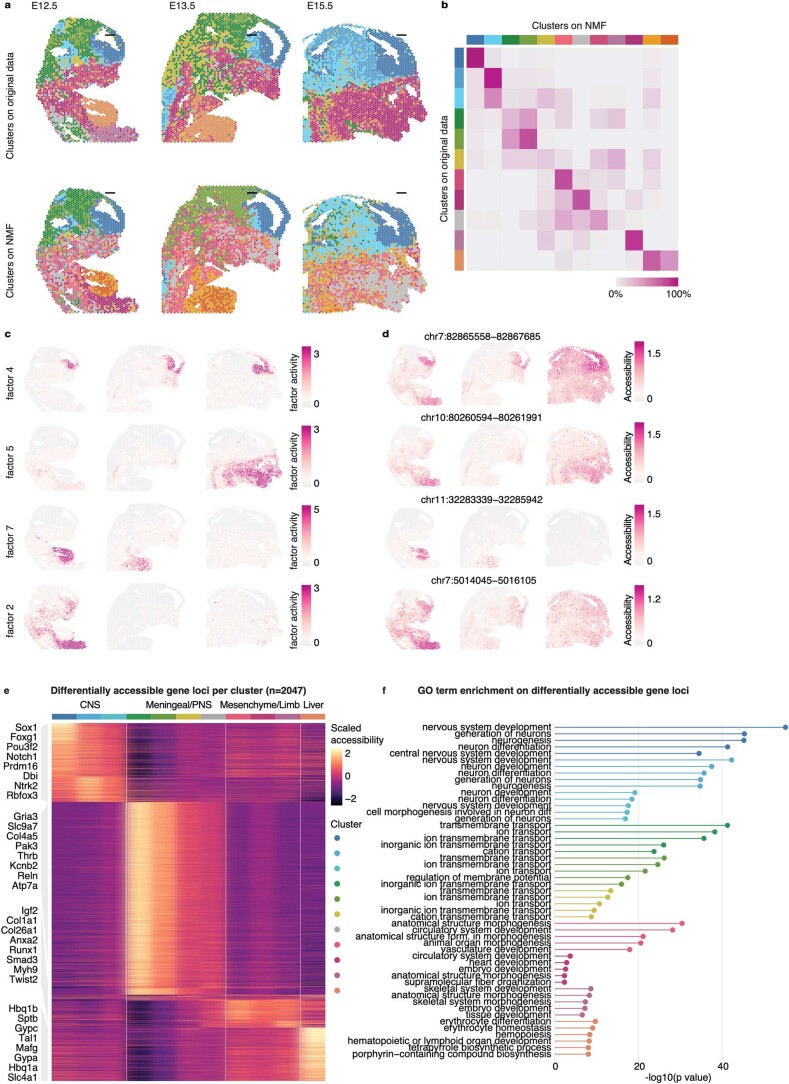
Clustering of spatial ATAC data with spatially aware factor analysis, marker genes and gene ontology analysis. **a**. Spatial ATAC clusters based on LSI (top; sections and clusters from Fig. 1c shown again for comparison) or NMF (bottom) for dimensionality reduction. **b**. Heatmap displaying the percentage of spots assigned to LSI- or NMF-computed clusters. **c**. Spatial activity plots for selected factors enriched in forebrain, facial prominence, liver, and limb. **d**. Examples of the most contributing peaks for each factor represented in c. Scale bars are 500 µm. **e**. Heatmap showing scaled accessibility for the top differentially accessible genes (gene body + promoter) across clusters. Relevant markers are highlighted. **f**. Gene ontology enrichment analysis of the top marker genes colored by cluster. *P* values were determined with hypergeometric test followed by correction for multiple testing using g:Profiler’s g:SCS algorithm.

We performed spatial ATAC on replicate tissue sections from three stages of mouse gestational development (embryonic days E12.5, E13.5 and E15.5). Spatially barcoded open chromatin fragments showed high enrichment around transcriptional start sites (TSS), as well as nucleosome periodicity, hallmarks of ATAC-seq (Fig. 1b and Extended Data Fig. 1). We captured a median of 6,100, 3,100 and 7,100 unique fragments per 55 µm spot, with 14, 15 and 18% overlapping TSS in E12.5, E13.5 and E15.5 sections, respectively. These metrics are comparable with published single-nucleus and microfluidics-based spatial ATAC-seq data from the developing mouse (Extended Data Fig. 1a–c). Additionally, the aggregate distribution of fragments across the genome showed a very high concordance with reference single-nucleus ATAC-seq (snATAC-seq) datasets from the Encyclopedia of DNA Elements (ENCODE)^14^ (Extended Data Fig. 1d,e). We next created a peak-spatial barcode count matrix using a common reference peak set across sections that were analyzed by latent semantic indexing (LSI) and uniform manifold approximation and projection (UMAP) for dimensionality reduction^15^. Unsupervised clustering identified 11 main clusters, which revealed high concordance with anatomical landmarks when projected onto their original spatial coordinates and were consistent, not only across replicate sections, but also across developmental stages and analytical strategies (Fig. 1c,d and Extended Data Figs. 2 and 3). This clustering further agreed with spatially aware non-negative matrix factorization dimensionality reduction and clustering^16^, suggesting that spatial location is a major source of variation in chromatin accessibility across and within developing tissues (Extended Data Fig. 4a–d). As expected, the dataset structure reflected variation in the accessibility of promoters and a larger set of distal peaks (Fig. 1e). Using differential accessibility analyses we found 18,000 differentially accessible peaks that showed specific patterns of accessibility across developing tissues (Fig. 1f, g).

**Extended Data Fig. 5:**
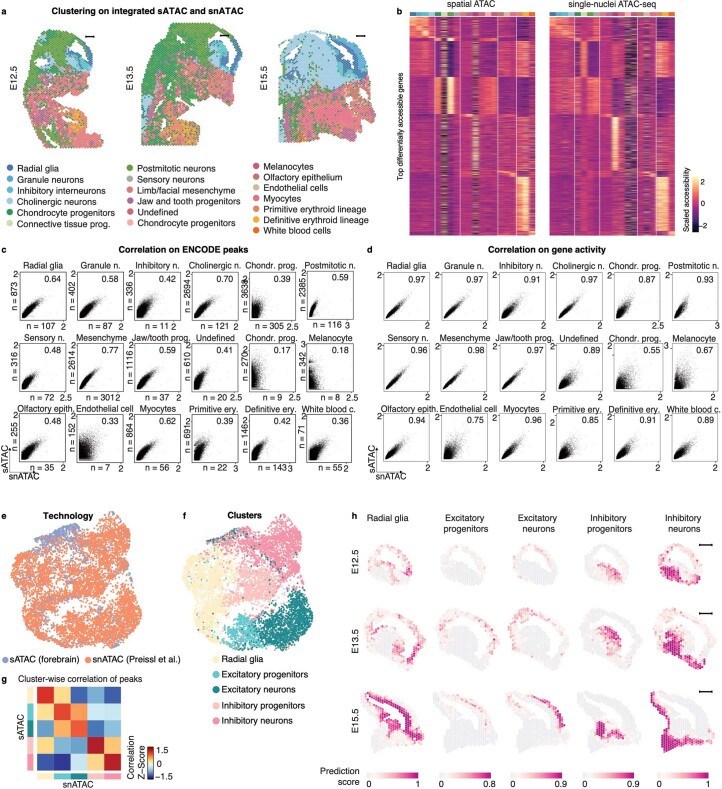
Integration of spatial ATAC with single-nucleus ATAC-seq (snATAC-seq) during mouse development. **a**. Representative spatial ATAC sections with clustering based on integration with snATAC-seq data from the same developmental stages (clusters and colors consistent with Fig. 1j, k). **b**. Heatmaps showing scaled accessibility for differentially accessible gene loci (n = 2575) for all clusters across both technologies. **c**. Scatterplots comparing log-normalized accessibility at ENCODE peaks across clusters and technologies. Spearman’s correlation coefficients are shown inside the plot and spot/cell numbers are reported in the respective axes. **d**. Same as in d but for log-normalized gene activity counts. **e**. UMAP embedding for spatial ATAC forebrain spots and age-matched snATAC-seq data from^14^ colored by technology. **f**. UMAP embedding colored by cell identity according to Preissl et al. **g**. Heatmap depicting z-scored correlation coefficients for accessibility at ENCODE peaks across clusters and technologies. **h**. Prediction scores for snATAC-seq-defined cell clusters in f on spatial ATAC forebrain regions across developmental stages.

We next computed gene activities (that is, accessibility at gene locus and promoter), which revealed 2,000 differentially accessible genes between clusters that were enriched for gene ontology terms characteristic of the respective tissue region (Extended Data Fig. 4e, f). For example, central nervous system clusters showed increased accessibility in genes known to be involved in neurogenesis (for example, *Sox1*, *Foxg1*, *Notch1*). Bone and muscle mesenchyme clusters showed increased accessibility in myofiber, collagen and TGF-b signaling genes (for example, *Myh9*, *Col1a1*, *Smad3*), while the fetal liver cluster was characterized by accessibility of genes involved in erythropoiesis (for example, *Hba-a1*, *Tal1*, *Sptb*). We next generated snATAC-seq profiles from matched developing embryos for direct comparison. Spatial ATAC spots integrated well with snATAC-seq data, which further increased clustering granularity within tissue structures (Fig. 1h–k). Genome-wide chromatin accessibility correlation across cell types was high between technologies, which allowed us to accurately predict the spatial location of individual cells (Extended Data Fig. 5).

**Fig. 2:**
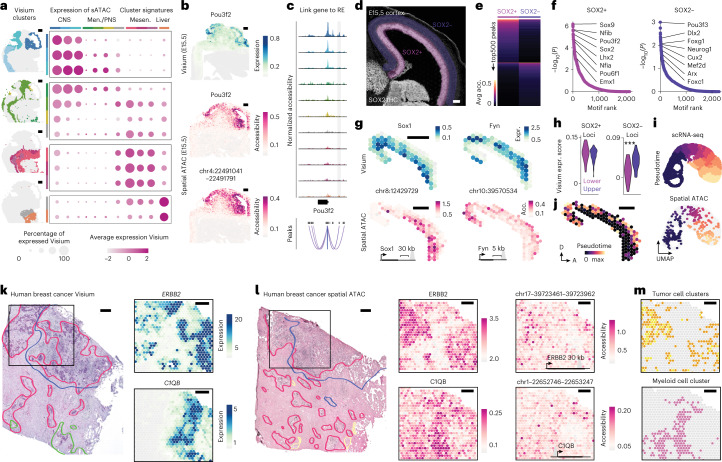
Spatial ATAC uncovers spatiotemporal patterns of regulatory element accessibility underlying gene expression. **a**, Visium gene expression signature scores for differentially accessible genes in spatial ATAC (sATAC) clusters. Visium clusters (left) on an E12.5 section for reference. CNS, central nervous system; Men./PNS, meninges/peripheral nervous system; and Mesen., mesenchyme. **b**, *Pou3f2* expression (top, cyan), gene activity and accessibility of a co-accessible distal regulatory element (magenta). **c**, Genomic track and co-accessibility scores for peaks around the *Pou3f2* locus. The distal element shown in **b** is highlighted in gray and tracks are colored according to spatial ATAC clusters. **d**, Inset of a SOX2-immunostained E15.5 spatial ATAC section (*n* = 2) with highlighted SOX2+ (progenitor, pink) and SOX2− (neuronal, purple) regions. **e**, Top 500 differentially accessible peaks by fold change in SOX2+ and SOX2− regions. Avg. acc., average accessibility. **f**, Motif enrichment analysis of peaks from **e**. Selected motifs for transcription factors expressed in the region are highlighted. *P* values by a one-sided hypergeometric test. **g**, Accessibility (Acc.) (spatial ATAC; magenta) and expression (expr.) of the nearest gene (Visium; cyan) for loci enriched in progenitor (*Sox1*) or neuronal (*Fyn*) regions. **h**, Gene signature score in lower and upper cortical regions for differentially accessible genes in SOX2+ and SOX2− regions. **i**, UMAP of integrated single-cell RNA-seq and spatial ATAC from the E15.5 developing cortex colored by pseudotime and split by technology. *P* values by Wilcoxon test (***<0.001). **j**, Pseudotime scores projected onto their spatial locations in a spatial ATAC E15.5 section. **k**, Hematoxylin and eosin image of a breast cancer section processed using Visium (*n* = 1) with overlaid pathologist annotations. Expression of *ERBB2* (HER2) and myeloid cell marker *C1QB* in the boxed inset. **l**, Annotated hematoxylin and eosin image of an adjacent (200 µm) section processed using spatial ATAC (*n* = 3). On the right, accessibility of the *ERBB2* locus, *C1QB* locus and two associated regulatory regions in the boxed inset (right). **m**, Spatial interaction between tumor cell and myeloid cell clusters at the tumor interface. Pathology is denoted as follows: red, invasive cancer; blue, tumor infiltrating lymphocytes; green, intravascular cancer and yellow, normal gland. Scale bars, 500 µm.

**Extended Data Fig. 6:**
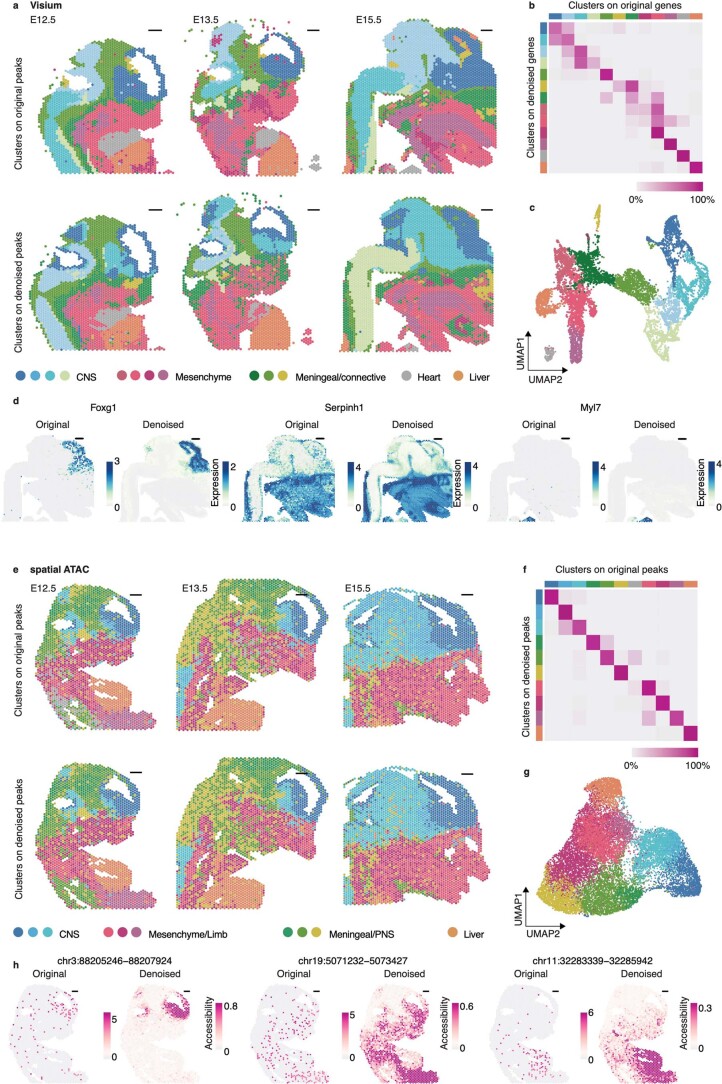
Deep count autoencoder denoising of Visium and spatial ATAC. **a**. Visium clusters based on original (same as in Fig. 2a) or denoised gene counts. **b**. Heatmap displaying the percentage of spots being assigned to the clusters obtained from original or denoised Visium data. **c**. UMAP on denoised expression colored by cluster. **d**. Visualization of gene expression normalized counts before and after denoising on E15.5 sections. **e**. Clusters based on original or denoised spatial ATAC peak counts (same as in Fig. 1c, shown for comparison). **f**. Heatmap displaying the percentage of spots being assigned to the clusters obtained from original or denoised spatial ATAC data **g**. UMAP on denoised peaks colored by cluster. **h**. Visualization of normalized peak accessibility before and after denoising on E12.5 sections. Scale bars are 500 µm.

**Extended Data Fig. 7:**
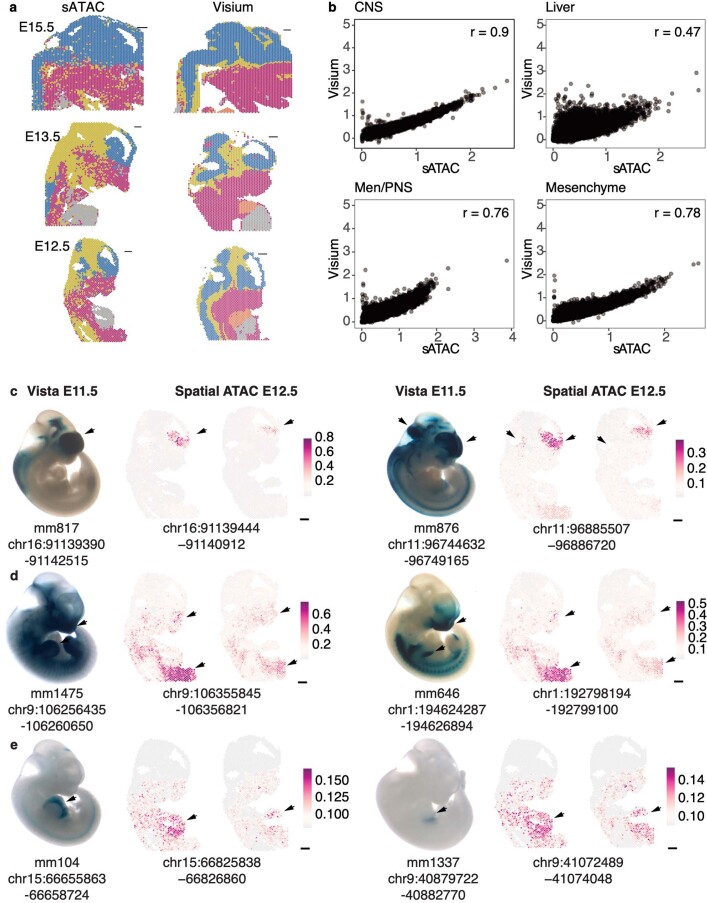
Correlation between accessibility and expression and validation of spatial ATAC regulatory element accessibility. **a**. Spatial ATAC and Visium with clusters grouped according to the main anatomical structures common to the sections. **b**. Scatterplots showing the log-normalized counts for Visium gene expression and spatial ATAC gene activities across anatomical structures. Correlation coefficient is shown in the plot. **c**. Vista enhancer reporter expression for two CNS elements overlapping with differentially accessible spatial ATAC peaks. Genome coordinates under the Vista image according to mm9 genome assembly. Under spatial ATAC feature plots, mm10. **d**. Vista enhancer reporter expression for two liver elements overlapping with differentially accessible spatial ATAC peaks. **e**. Vista enhancer reporter expression for two limb elements overlapping with differentially accessible spatial ATAC peaks. Reporter images were obtained from https://enhancer.lbl.gov/. Scale bars are 500 µm.

**Extended Data Fig. 8:**
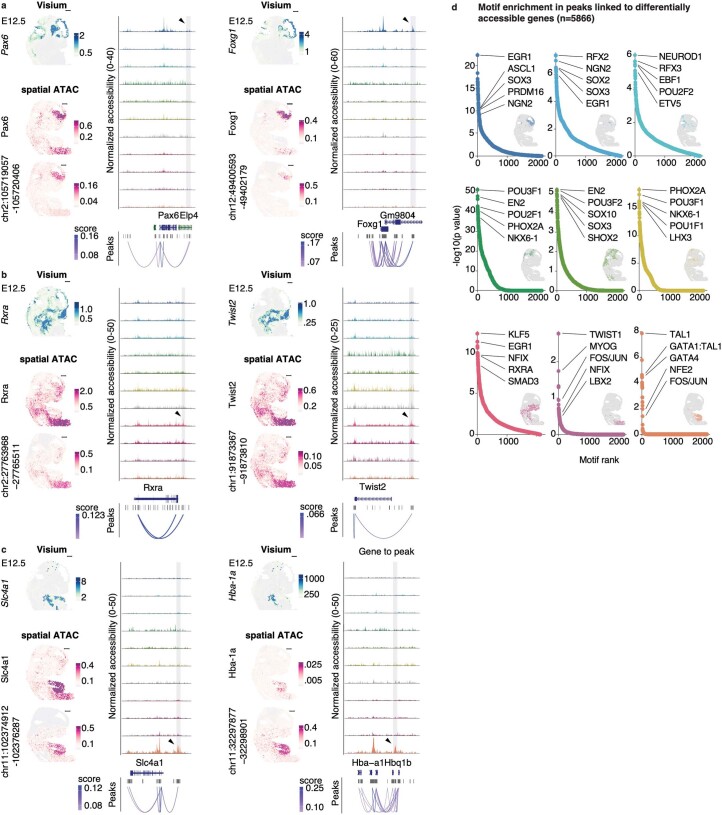
Gene regulatory programs during mouse organogenesis. **a**. Visium expression, spatial ATAC gene activity and regulatory element accessibility at E12.5 for CNS/Forebrain markers Pax6 and Foxg1. The respective linked regulatory element is shown in gray. **b**. Visium expression, spatial ATAC gene activity and regulatory element accessibility for Mesenchyme/Limb markers Rxra and Twist2. The respective linked regulatory element is shown in gray. **c**. Visium expression, spatial ATAC gene activity and regulatory element accessibility for liver markers Slc4a1 and Hba-a1. The respective linked regulatory element is shown in gray. Arrowheads point to clusters for which the regulatory element is most accessible. **d**. Motif enrichment rank plots for cluster-specific distal elements. Selected top non-redundant transcription factor motifs are highlighted. *P* values were determined by a one-sided hypergeometric test. Scale bars are 500 µm.

Next, we sought to integrate spatial ATAC with Visium spatial transcriptomics. We performed Visium on tissue sections from the same developmental stages, which showed regionally consistent clustering and genes found as differentially accessible using spatial ATAC showed higher expression in the corresponding Visium cluster (Fig. 2a). Unsupervised denoising and imputation methods have been developed to account for the intrinsic sparsity of single-cell transcriptomics and ATAC-seq data that improve visualization and feature-to-feature correlation^17,18^. We applied a denoising deep count autoencoder (DCA) to our spatial ATAC and Visium datasets^18^, which increased the signal-to-noise ratio in feature visualizations while preserving clustering structure (Extended Data Fig. 6). Accessibility at gene loci correlated with gene expression across anatomical structures (Extended Data Fig. 7). To identify putative regulatory elements underlying spatial patterns of gene expression, we performed peak co-accessibility analyses for differentially accessible gene loci. With this strategy, we identified 6,000 individual distal regulatory elements whose accessibility correlated to gene expression across tissues (Extended Data Fig. 8) and agreed with enhancer reporter assays (Extended Data Fig. 7c–e). To gain further insight into regulatory programs underlying gene expression, we performed motif enrichment analysis on these cluster-specific distal peaks. We found that the most enriched motifs in central nervous system clusters corresponded to well-characterized proneural transcription factors (for example, Neurog1, Neurod1, Ascl1). Conversely, motifs enriched in mesenchymal regulatory elements corresponded to factors known to be involved in bone and muscle development (for example, Smad3, Twist1, Myog), while liver-specific distal regulatory elements were highly enriched in binding sites for Tal1 and Gata transcription factors, consistent with their role in hematopoiesis (Extended Data Fig. 8d).

To evaluate whether spatial ATAC could identify regulatory programs underlying lineage differentiation within a developing tissue, we focused on the cerebral cortex at E15.5, a well-characterized structure in which SOX2+ progenitors in the subventricular zone generate neurons that migrate to upper cortical layers^19^. Based on SOX2 immunostaining, we selected progenitor- and neuron-rich spots and performed motif enrichment on the top differentially accessible peaks (Fig. 2d–f). We identified cortical progenitor (for example, Sox2, Lhx2, Emx1) and neuronal (for example, Neurog1, Cux2) transcription factors among the top enriched motifs in the respective clusters (Fig. 2f). Further, we could link regulatory elements to the nearest genes that showed the corresponding patterns of layer-specific gene expression, and gene accessibility correlated with expression in the respective cortical layer (Fig. 2g,h). Next, we integrated the cortical spatial ATAC spots with single-cell RNA-sequencing (scRNA-seq) data from the same developmental stage^20^. Using the integrated dataset, we calculated pseudotime scores along the neuronal differentiation trajectory, which aligned single cells and spatial ATAC spots and recapitulated the inside-out differentiation trajectory of the developing cortex (Fig. 2i–j).

**Extended Data Fig. 9:**
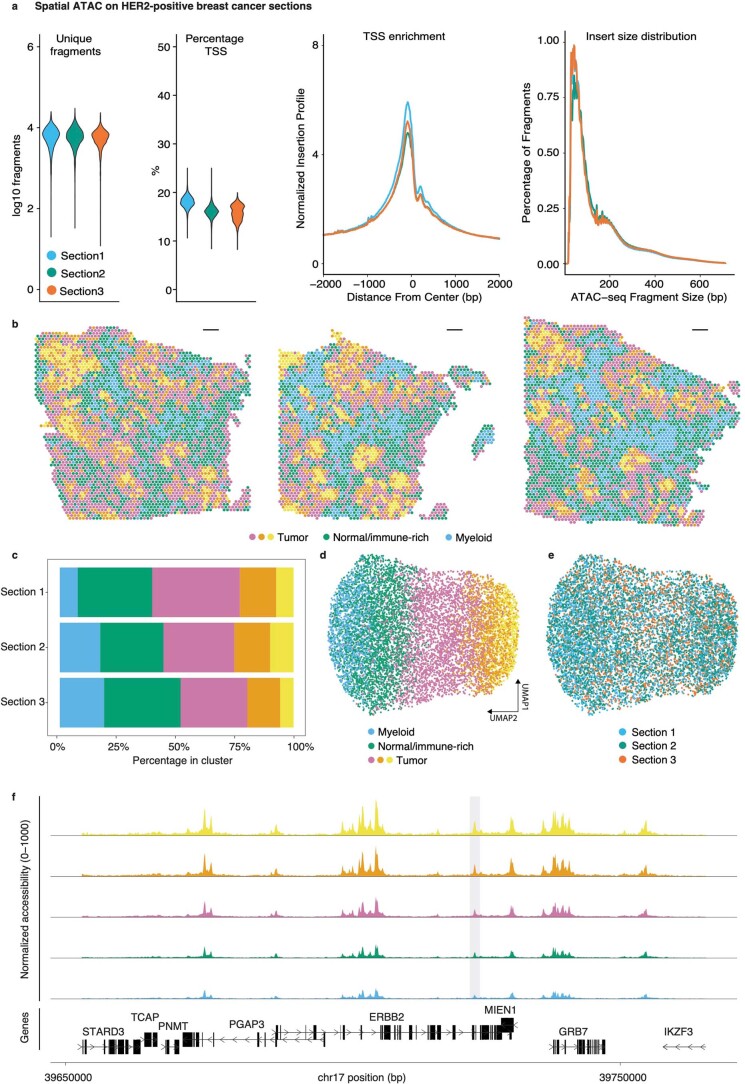
Spatial ATAC on human HER2-positive breast cancer. **a**. On the left, violin plot showing unique fragments per spot and percentage TSS fragments in three adjacent sections processed using spatial ATAC. On the right, TSS enrichment and insert size distribution. **b**. Spatial ATAC clustering reveals tumor, immune-rich and normal tissue regions. **c**. Cluster percentage across sections. **d-e**. UMAP embedding on spatial ATAC peaks color-coded by cluster or tissue section. **f**. Genome tracks showing normalized spatial ATAC-seq fragment density around the HER2 (ERBB2) locus colored by cluster. The gray area marks a previously described gene body enhancer^36^ shown in Fig. 2l. Scale bars are 500 µm.

**Extended Data Fig. 10:**
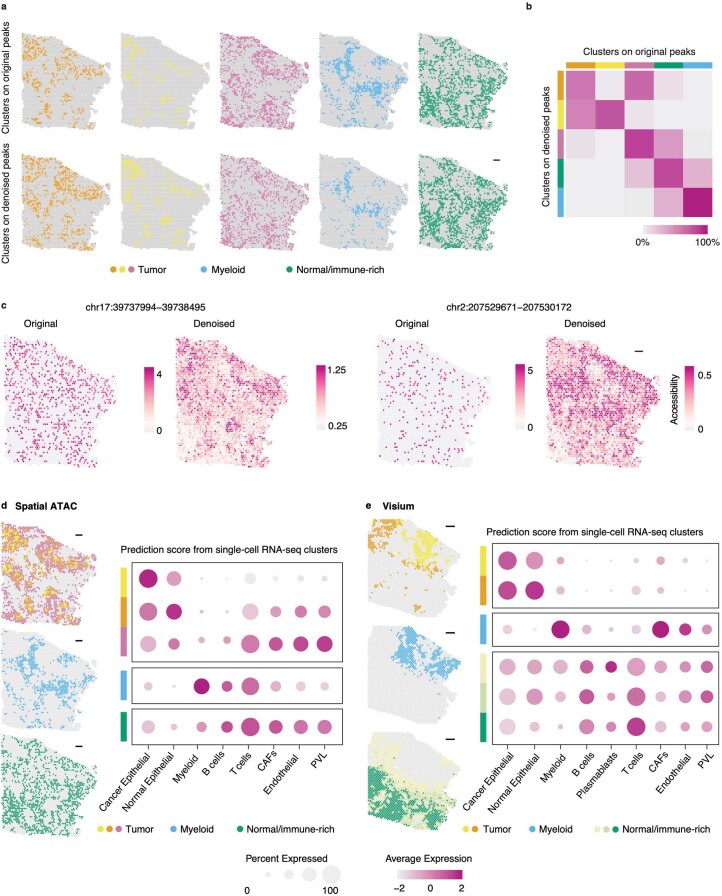
Multimodal integration of single-cell RNA-seq with spatial ATAC and Visium on breast cancer sections. **a**. Clusters based on original or DCA denoised peak counts. **b**. Heatmap displaying the percentage of spots being assigned to the clusters obtained from original or denoised spatial ATAC data. **c**. Visualization of peak accessibility scores before and after denoising on one example section. **d**. Prediction scores from scRNA-seq signatures in spatial ATAC clusters reveal cell composition differences across clusters. **e**. Prediction scores from scRNA-seq signatures in Visium clusters. Scale bars are 500 µm.

Finally, we applied spatial ATAC to human breast cancer, a tumor type of widespread public health concern in which pathological classification informs therapy decisions^21^. We profiled adjacent sections using Visium and spatial ATAC. Spatial ATAC clustering and marker expression aligned with pathologist annotations, agreed with Visium clustering and could readily identify HER2-positive regions, their associated non-coding region accessibility and the presence of myeloid cells in the immediate tumor microenvironment (Fig. 2k–m and Extended Data Figs. 9 and 10).

Our spatial ATAC platform is readily implementable through common laboratory workflows and offers the possibility for integration with other existing and future ‘omics’ modalities. We envision that spatial ATAC will enable spatial non-coding functional genomics, while being instrumental in the identification of regulatory elements for specific cell targeting in gene therapy and the study of gene regulatory networks in development and disease.

## Methods

### Animal tissue processing

Time pregnant C57BL/6 mice were purchased from Janvier and were euthanized by cervical dislocation at embryonic day 12.5, 13.5 or 15.5 for embryo harvesting. All experimental procedures were carried out in accordance with the Swedish and European Union guidelines and approved by the local committee for ethical experiments on laboratory animals in Sweden (Stockholms Norra Djurförsöksetiska Nämnd) under ethical permit numbers N155/16 and 20785/2020.

The tissues were harvested on ice-cold PBS and snap frozen in optimal cutting temperature compound (Tissue-Tek, 4583) blocks in a dry ice-isopentane bath at −60 °C and stored at −80 °C until being sectioned.

### Collection of tumor samples from patients with breast cancer

Breast cancer tissues were obtained from the Department of Clinical Pathology and Cancer Diagnostics at Karolinska University Hospital, Stockholm, Sweden. Experimental procedures and protocols were approved by the regional ethics review board (Etikprövningsnämnden) in Stockholm (2016/957-31, amendments 2017/742-32 and 2021-00795), and informed consent was obtained from the participating patient.

The samples were obtained from a breast tumor removed from a patient with treatment-naive invasive ductal carcinoma. The tumor was divided into several regions and collected freshly by a pathologist depending on the size of the tumor. From each region, tissue was isolated for direct embedding in optimal cutting temperature compound, followed by immediate freezing and storage at −80 °C until further analysis. Histological evaluations of the patient’s tumor were performed by pathologists for diagnostic purposes: tumor characteristics, including grade, size, hormone receptor, HER2 and KI67 status are presented in Supplementary Table 3.

### Spatial ATAC

Cryosections were cut on a cryostat (Leica, NX70) at a 10 μm thickness and placed on spatially barcoded OMNI glass slides (10X Genomics). In brief, each OMNI array slide contained eight capture areas, each covered by 5,000 barcoded spots with diameters of 55 and 100 μm between spots. Each spot contained millions of DNA oligonucleotides encoding a 16 nt spatial barcode, serving as *x* and *y* coordinates, a PCR handle for library amplification, a 12-nt unique molecular identifier and a 7-nt generic capture sequence used for splint oligonucleotide hybridization (Supplementary Table 1). Slides were first heated at 37 °C for 1 min to adhere the tissue to the slide. Then, the sections were crosslinked in freshly prepared methanol-free 0.5% formaldehyde (Polysciences, 18814) diluted in Dulbecco’s PBS (DPBS) for 10 min at room temperature, followed by rinsing in 500 mM Tris-HCl pH 8 (Thermo, AM9856) to quench the formaldehyde. After dipping the slide in DPBS three times, the sections were immunostained as follows: the tissue sections were blocked by incubation for 5 min with staining buffer (DPBS containing 5% Donkey serum, 0.1% NP-40 (Thermo 28324) and 0.005% Digitonin (Promega G9441)). The staining buffer was then removed, and the primary antibody dilution added (antibodies used were: rabbit anti-SOX2 Merck 5603, 1:100; goat anti-SOX9 R&D 3075, 1:300 and antinuclear antigen Novus 235-1, 1:100) and incubated at room temperature for 30 min. Then, washing was performed twice with staining buffer for 3 min each, followed by addition of donkey anti-rabbit or anti-goat Alexa 647-conjugated IgG secondary antibodies (Thermo 31573 or 21447; 1:500), and incubation at room temperature for 15 min. Then, washing was performed three times with staining buffer for 3 min each and finally pipette-washed with DPBS once. The slides were then spin dried, covered with 85% glycerol, mounted with a coverslip and imaged in a Zeiss LSM 700 (×10 magnification) confocal or in a Metafer VSlide system (×20 magnification) epifluorescence microscope to record tissue coordinates and capture area fiducials. The images were processed with the VSlide software (v.1.0.0) or with Fiji (v.2.3.0)^22^.

After image acquisition, the glycerol was removed by dipping in DPBS and a layer of isopropanol was then added to the arrays, decanted and air-dried. The slide was then rehydrated in DPBS followed by ATAC permeabilization (0.01% digitonin, 0.1% Tween-20, 0.1% NP-40, 10 mM Tris-HCl pH 7.4, 10 mM NaCl, 3 mM MgCl_2_) at room temperature for 10 min.

Custom Tn5 transposomes (30 μM) were assembled using Nextera adapter oligonucleotides A and B (Supplementary Table 1) according to ref. 23. Tagmentation was performed according to OMNI ATAC-seq^24^ at 37 °C for 1 h under gentle shaking (300 rpm every 5 min) using 2 μl Tn5 in tagmentation mix (25 μl 2× TD buffer, 16.5 μl DPBS, 0.5 μl 1% digitonin, 0.5 μl 10% Tween-20). To stop the tagmentation and strip the transposase from DNA, sections were incubated with 50 mM EDTA while ramping down to 30 °C for 10 min. To hybridize the tagments to the barcoded surface oligonucleotides, we then incubated the sections with a 2 μM solution of splint oligonucleotide (in 3× SSC buffer containing 0.01% Triton-X100, 0.8 μg μl^−1^ Proteinase K and 2.5% PEG8000) overnight at 30 °C. Next, the sections were rinsed in 2× NEB 2.1 buffer, and subsequently incubated with ligation and polymerization solution (1× NEB 2.1 containing 3 U of T4 DNA polymerase, 2,000 U of T4 DNA ligase, 100 μM dNTPs, 1 mM ATP, all from NEB) and incubated at 18 °C for 4 h. Tissue removal was then performed using 2 mg ml^−1^ Proteinase K in PKD-buffer (Qiagen), and incubated at 56 °C for 30 min (shaking at 300 rpm). The slides were then sequentially washed in 2× SSC 0.1% SDS, 0.2× SSC and 0.1× SSC and finally spin dried.

### Library preparation and sequencing

Spatially barcoded single-stranded DNA fragments were released from the surface by denaturation with 0.08 M KOH at room temperature for 10 min and then quenched in 10 μl of 1 M Tris pH 7. The denatured fragments were pH adjusted with sodium acetate and cleaned with MinElute Reaction Cleanup Kit (Qiagen, 28204). The eluted DNA was then amplified using PCR using Partial.R1 and Ad2.short oligonucleotides for 15 cycles using PrimeSTAR Max DNA Polymerase mix (Takara, R045B). The amplified products were purified using 0.9× SPRI beads and i7-indexed in a second PCR (four cycles) using PE1.0 and Ad2.X (where X is the sample index from ref. 12) oligonucleotides and KAPA HiFi HotStart Mix (Roche, KK2602). The final indexed libraries were cleaned up using 0.8× SPRI beads and adjusted to the desired molarity based on the concentrations measured using Qubit HS double-stranded DNA Assay Kit (Thermo, Q32854) and the average fragment size from HS DNA Bioanalyzer kit (Agilent, 5067-4626).

Pooled libraries were then sequenced on Illumina Nextseq 550 or 2000 instrument using custom sequencing oligonucleotides for Read1 and Index2 (CustomR1 and CustomI2). We sequenced 65 bases for reads 1 and 2 (genomic sequence), 28 bases for i5 (spatial barcode and unique molecular identifier) and eight bases for i7 (sample index). All DNA oligonucleotides are listed in Supplementary Table 1.

### H&E staining

Tissue sections from breast cancer specimens were first dried with isopropanol (Fisher Scientific, A461-1) before staining. The sections were then stained with Mayer’s hematoxylin (Agilent, S3309) for 4 min, washed in ultrapure water, incubated in bluing buffer (Agilent, CS702) for 2 min, washed in Milli-Q water and further incubated for 1 min in 1:20 eosin solution (Sigma-Aldrich, HT110216) in Tris-buffer (pH 6). The tissue sections were dried for 5 min at 37 °C and then mounted with 85% glycerol (Merck, 104094) and a coverslip. Imaging was performed using the Metafer VSlide system at ×20 magnification.

### Data preprocessing

Raw reads were preprocessed using 10X Genomics’ CellRanger ATAC pipeline (v.2.0.0). We used a custom ‘barcode_whitelist’ specifying positional barcodes from the spatial arrays and default reference genomes (mm10, v.2.0.0 for the mouse data and hg38, v.2.0.0 for the human data). All other parameters for ‘mkfastq’ and ‘count’ functions were set to default. Sequencing data from each section was processed separately and subsequently integrated with Seurat (v.4.1.0, ref. 25) and Harmony (v.0.1.0, ref. 26) R packages (below).

### Analysis and visualization

For the embryos, we assayed sections across different developmental stages and integrated them for downstream analysis. To do so, we first obtained age-specific fragment files from ENCODE^27^ and merged them using GenomicRanges’s (v.1.46.1, ref. 28) ‘reduce()’ function. We then used these to create new accessibility matrices with a common set of 269,767 peaks. For comparison, we also called peaks on the merged dataset using MACS2 (v.2.2.6), as well as constructed feature matrices from 5 kb genomic bins, and inspected the clustering concordance across pre-processing strategies. Peak-barcode matrices for the human breast cancer sample were constructed using a set of 215,978 peaks from ref. 4. We next subset the matrices to only include spots overlaying tissue, which were manually identified in Loupe Browser (v.6.0.0) after aligning immunofluorescence pictures with capture area fiducials. Loupe browser was also used to select SOX2+ and SOX2− cortical spots in two mouse E15.5 sections. The spatial object was created using STutility R package (v.0.1.0, ref. 16), using tissue spot coordinates adjusted for the dimensions of the microscope images. STutility was used to produce the spatial plots using ‘ST.FeaturePlot()’ function for quantitative variables. TSS enrichment plots and FragmentHistograms were generated using ArchR (v.1.0.1)^29^.

For each tissue type, we merged sections and performed normalization and dimensionality reduction on all peaks using Signac’s (v.1.6.0, ref. 15) ‘RunTFIDF()’ and ‘RunSVD()’ functions with default settings. We calculated gene activity using Ensembl annotations (EnsDb.Mmusculus.v79, v.2.99.0 and EnsDb.Hsapiens.v86, v.2.99.0), followed by log-normalization and principal component analysis. Genes from the *Pcdh* and *Ugt* gene clusters were removed from the gene activity assay before downstream analysis. For the embryos, graph clustering and UMAP were then performed on the peaks assay after integrating section-wise with Harmony on the top seven dimensions and at a resolution of 0.7, which enabled identification of clusters that reflect the underlying anatomical structures. Human breast cancer sections, which were obtained from the same tissue specimen, were merged directly using Seurat’s ‘merge()’ function followed by UMAP and graph clustering on dimensions 2 to 7, and at a resolution of 0.5. Cluster-wise Spearman’s correlation of the chromatin accessibility profile was calculated for peaks around the transcription start site (that is, between −1,000 bp and +100 bp from TSS position) and for distal elements, using GenomicRanges’s GetTSSPositions() followed by Signac’s ClosestFeature() functions to annotate the peaks, and Seurat’s ‘AverageExpression()’ to obtain cluster-wise average accessibility levels for each peak. Differential accessibility analysis was carried out on peaks using Seurat’s FindAllMarkers() function with method = ‘LR’ and unique fragments as the latent variable, and with logfc.threshold = 0.2 and min.pct = 0.01 to account for the sparsity of ATAC-seq data. FindAllMarkers() was also ran on the gene activity data with Wilcoxon’s Rank Sum test and followed by Gene Ontology analysis using gprofiler2 R package (v.0.2.1). Differentially accessible features were retained at an adjusted *P* value of 0.05 after Bonferroni’s correction. Co-accessible peaks were identified after running LinkPeaks() on differentially accessible genes with a correlation cut-off, as well as a minimum 1 kb distance from the TSS. Motif enrichment analysis was carried out using FindMotifs() function and a set of clustered motifs from ref. 30 on all linked peaks. Non-redundant top motifs were highlighted. For motif enrichment analyses in the developing mouse cortex, we first ran FoldChange() on peaks from SOX2+ and SOX2− cortical spots and then selected the top 500 peaks for motif analyses as above. Full lists of enriched motifs are provided in Supplementary Table 2. Vista enhancers were downloaded from https://enhancer.lbl.gov/ and genome coordinates were lifted to mm10 using the UCSC liftover tool before intersection with spatial ATAC tissue-specific peaks using bedtools (v.2.19.0)^31^.

### Denoising

Using a DCA (v.0.3.4, ref. 18), we denoised the peak-barcode matrix of the combined objects, as well as the gene activity matrices. For the peaks data, we specified the following parameters: –nosizefactors –nonorminput –nologinput, whereas DCA was run with default settings on the gene activity data. Additionally, we performed DCA with default parameters on Visium data from the mouse embryo and human breast cancer (below). Dimensionality reduction and clustering was performed on the denoised data as above to evaluate concordance between original and denoised data. While clustering and differential accessibility analysis were conducted on original data, denoised data was used for visualization of accessibility levels and for multimodal integration with single-cell data (below).

### Spatial analysis

STutility’s RunNMF() function was run with ‘nfactors = 8’ after ordering the top 25% variable features according to spatial correlation. Harmony integration on tissue section and graph clustering was performed using non-negative matrix factorization factors in dimensionality reduction and the groups obtained this way were compared with the spatial-agnostic clusters obtained with the original peaks assay.

### snATAC-seq

To analyze spatial ATAC datasets in conjunction with snATAC-seq, we prepared single nuclei suspensions from fresh frozen embryos (E12.5, E13.5 and E15.5) that were littermates to those used for spatial ATAC. Three to five 70 µm frozen sections were obtained for each embryonic stage matching the anatomical landmarks from spatial ATAC sections. Frozen sections were then dissociated according to the 10X Chromium Single Nuclei Isolation kit (1000494) omitting the debris removal step to avoid cell loss. Nuclei suspensions were stained with 7-AAD (Miltenyi; 1:50) and sorted on a BD Fusion flow cytometer with a 100 µm nozzle. Nuclei were then immediately processed according to the 10X Genomics’ Single Cell ATAC Next GEM kit (v.1.1). Sequencing data were demultiplexed and mapped using CellRanger ATAC with default parameters yielding a total of 1,879 cells. Accessibility matrices were constructed with Signac’s FeatureMatrix() function using the ENCODE peak set to enable direct comparison with the spatial data. Single-nucleus data were subsequently integrated with the spatial profiles using FindIntegrationAnchors() with ‘rlsi’ reduction, followed by IntegrateEmbeddings() and RunHarmony() with sample of origin as grouping variable, which was used to obtain UMAP visualizations of the co-embedded data. The concordance of spatial and single-nucleus chromatin accessibility data was subsequently explored by cluster-wise correlation analysis of all peaks and gene bodies that were log-transformed and normalized to adjust for sequencing depth. Differential accessibility testing for gene activities was used for cluster annotation using ref. 17 for reference. Furthermore, we mapped the clusters resulting from integration onto the spatial ATAC sections to confirm the validity of our annotations.

We further analyzed our spatial ATAC data together with published snATAC-seq profiles of forebrain development sampled at the same developmental stages (that is, E12.5, E13.5 and E15.5). For this purpose, we constructed accessibility matrices from the snATAC-seq^10^ data using the ENCODE peaks set, and using the Loupe Browser we subset the spatial ATAC profiles to only include capture spots overlaying the forebrain. Next, we integrated the multimodal data as above and calculated prediction scores on the spatial data for each of the clusters in the snATAC-seq profiles (that is, by means of Signac’s TransferData() function).

### Visium

The 10X Genomics’ Visium platform was used to obtain spatial transcriptomics data for tissue samples matching our spatial ATAC sections (that is, either on consecutive tissue slices from breast tumor block or on similar sagittal level of embryos from the same litter).

Raw data were pre-processed using SpaceRanger’s (v.1.3.1) mkfastq and count functions with default parameters, and the resulting gene-barcode matrices were then analyzed with Seurat for normalization, dimensionality reduction and clustering, and with STUtility for plotting. Visium data were denoised with DCA and default parameters for visualizations and comparison with spatial ATAC data.

### Integrative multimodal analysis

We performed multimodal comparison of our spatial ATAC data using either spatial or single-cell transcriptomics. To measure cluster-wise concordance between gene expression and accessibility, we analyzed in parallel spatial ATAC and spatial RNA-seq data from the embryos and obtained cluster markers for each modality, which we used to calculate module scores (with Seurat’s AddModuleScore()) in each assay. Furthermore, we aggregated clusters into anatomical structures and performed correlation analysis between expression and accessibility of all genes in the dataset.

Additionally, we performed multimodal integrative analysis between spatial ATAC and single-cell RNA-seq data. For the embryos, we obtained a developmental transcriptional atlas from ref. 20, and subset it to include cells from E15 brains. In parallel, we restricted our analysis of spatial chromatin data to the cortex of E15.5 mice and manually subset spots overlaying the region of interest. Specifically, we focused our analysis to only comprise the dorsal forebrain and specifically looked at cells in the neurogenic trajectory (that is, radial glia, intermediate progenitors and neurons). Single-cell data were processed according to Seurat’s standard workflow and subset to *n* = 1,500 cells randomly sampled across the clusters. We integrated spatial ATAC and single-cell RNA-seq data using canonical correlation analysis and 2,000 anchor features. Co-embedded data were subsequently subjected to dimensionality reduction using principal component analysis. UMAP visualizations calculated on the top seven components were, finally, used to order cells in pseudotime using monocle3 (v.1.0.0, ref. 32) and the radial glia cluster as root cells.

For the human breast cancer data, we obtained a comprehensive single-cell RNA-seq atlas^21^ and processed it with Seurat’s standard workflow. We then probed enrichment of the main cell types in our spatial ATAC and spatial RNA-seq clusters. To do so, we adopted the author’s classification of cells in the highest tier (that is, ‘celltype_major’) and used Seurat’s label transfer workflow based on canonical correlation analysis to obtain prediction scores for each cell type in the single-cell dataset.

### Reporting summary

Further information on research design is available in the Nature Portfolio Reporting Summary linked to this article.

## Online content

Any methods, additional references, Nature Portfolio reporting summaries, source data, extended data, supplementary information, acknowledgements, peer review information; details of author contributions and competing interests; and statements of data and code availability are available at 10.1038/s41587-022-01603-9.

## Supplementary information

Reporting Summary

Supplementary Table 1 Oligonucleotide sequences.

Supplementary Table 2 Differentially accessible gene and peak sets. Motif enrichment analyses. Public datasets.

Supplementary Table 3 Patient data and tumor characteristics.

## Data Availability

All raw data and processed count matrices from mouse tissues can be obtained at Gene Expression Omnibus using the accession code GSE214991 (ref. 33). Human sequencing data are stored in the SciLife Data Repository at 10.17044/scilifelab.21378279.v1 (ref. 34). Additionally, we analyzed previously published datasets, a list of which is provided in Supplementary Table 2.

## Code availability

All analysis code used can be found at https://github.com/marzamKI/spatial_atac (ref. 35).
