# Supplementary material for: Solid-phase capture and profiling of open chromatin by spatial ATAC
Source: Nat Biotechnol. 2023 Jan 5;41(8):1085–8. doi: 10.1038/s41587-022-01603-9 (PMC10421738; doi:10.1038/s41587-022-01603-9)
Supplement: Supplementary file 1 — Reporting Summary [file 41587_2022_1603_MOESM1_ESM.pdf]

nature research

Corresponding author(s): Enric Llorens-Bobadilla
Patrik L Ståhl

Last updated by author(s): 2022/11/04

Reporting Summary

Nature Research wishes to improve the reproducibility of the work that we publish. This form provides structure for consistency and transparency in reporting. For further information on Nature Research policies, see our Editorial Policies and the Editorial Policy Checklist.

Statistics

For all statistical analyses, confirm that the following items are present in the figure legend, table legend, main text, or Methods section.

n/a	Confirmed	
☐	☒	The exact sample size (*n*) for each experimental group/condition, given as a discrete number and unit of measurement
☐	☒	A statement on whether measurements were taken from distinct samples or whether the same sample was measured repeatedly
☐	☒	The statistical test(s) used AND whether they are one- or two-sided<br>*Only common tests should be described solely by name; describe more complex techniques in the Methods section.*
☐	☒	A description of all covariates tested
☐	☒	A description of any assumptions or corrections, such as tests of normality and adjustment for multiple comparisons
☐	☒	A full description of the statistical parameters including central tendency (e.g. means) or other basic estimates (e.g. regression coefficient) AND variation (e.g. standard deviation) or associated estimates of uncertainty (e.g. confidence intervals)
☐	☒	For null hypothesis testing, the test statistic (e.g. *F*, *t*, *r*) with confidence intervals, effect sizes, degrees of freedom and *P* value noted<br>*Give P values as exact values whenever suitable.*
☐	☒	For Bayesian analysis, information on the choice of priors and Markov chain Monte Carlo settings
☐	☒	For hierarchical and complex designs, identification of the appropriate level for tests and full reporting of outcomes
☐	☒	Estimates of effect sizes (e.g. Cohen's *d*, Pearson's *r*), indicating how they were calculated

Our web collection on statistics for biologists contains articles on many of the points above.

Software and code

Policy information about availability of computer code

Data collection

Data was collected with an Illumina sequencer and software (v
Imaging data were acquired on a Metafer VSlide system and processed using VSlide software (v1.0.0)

Data analysis

Data analysis was performed with a combination of published packages and custom script, all of which can be found at https://github.com/marzamKI/spatial_atac
The softwares used in this analysis are:
CellRanger ATAC (v2.0.0)
Loupe Browser (v6.0.0)
SpaceRanger (v1.3.1)
MACS2 (v2.2.6)
DCA (v0.3.4)
R (v4.2.0)
bedtools (v2.19.0)
Fiji (v2.3.0)

The R packages used are:
Seurat (v4.1.0)
ArchR (v1.0.1)
Harmony (v0.1.0)
GenomicRanges (v1.46.1)
STutility R package (v0.1.0)
Signac (v1.6.0)
EnsDb.Mmusculus.v79 (v2.99.0)

EnsDb.Hsapiens.v86 (v2.99.0)
gprofiler2 R package (v.0.2.1)
monocle3 (v1.0.0)

For manuscripts utilizing custom algorithms or software that are central to the research but not yet described in published literature, software must be made available to editors and reviewers. We strongly encourage code deposition in a community repository (e.g. GitHub). See the Nature Research guidelines for submitting code & software for further information.

Data

Policy information about availability of data

All manuscripts must include a data availability statement. This statement should provide the following information, where applicable:

- Accession codes, unique identifiers, or web links for publicly available datasets
- A list of figures that have associated raw data
- A description of any restrictions on data availability

Raw data and processed count matrices generated from the mouse samples can be found using the accession code GSE214991.
Human sequencing data are stored in the SciLife Data Repository and can be accessed at 10.17044/scilifelab.21378279.
Additionally, we analyzed published datasets, which can be found in Table S2.

Reference genomes used to map mouse and human data were obtained by 10X Genomics as part of their standard Cellranger ATAC pipeline (v2.0.0).

Field-specific reporting

Please select the one below that is the best fit for your research. If you are not sure, read the appropriate sections before making your selection.

☒ Life sciences ☐ Behavioural & social sciences ☐ Ecological, evolutionary & environmental sciences

For a reference copy of the document with all sections, see nature.com/documents/nr-reporting-summary-flat.pdf

Life sciences study design

All studies must disclose on these points even when the disclosure is negative.

Sample size	No sample size calculations were performed. The sample size used in this study is in the same range as other studies using similar technologies. For embryo spatial ATAC data, data for each developmental age was acquired in 2 replicates (i.e. adjacent tissue sections from the same specimen). For the human spatial ATAC data, we processed 3 replicates. Spatial transcriptomics was performed on 1 or 2 sections from the same tissue.
Data exclusions	Spatial ATAC and transcriptomics data were inspected on Loupe browser and filtered through manual selection of capture areas that were not underlying the tissue slice. Gene activity matrices computed from spatial chromatin data were filtered to exclude the large genes clusters Pcdh and Ugt.
Replication	We performed different types of data processing, including producing feature-barcode matrices using an ENCODE consensus peak set or following MACS2 peak calling, as well as denoising with DCA. Data processed in different ways were then used to assess clustering concordance across tissues and replicates, which always showed good overlap.
Randomization	There was no randomization applied in this study. Embryos from different pregnant mice were used to acquire spatial transcriptomics and ATAC data.
Blinding	Clustering of data was performed using unsupervised approaches.

Reporting for specific materials, systems and methods

We require information from authors about some types of materials, experimental systems and methods used in many studies. Here, indicate whether each material, system or method listed is relevant to your study. If you are not sure if a list item applies to your research, read the appropriate section before selecting a response.

Materials & experimental systems

n/a	Involved in the study	
☐	☒	Antibodies
☒	☐	Eukaryotic cell lines
☒	☐	Palaeontology and archaeology
☐	☒	Animals and other organisms
☐	☒	Human research participants
☒	☐	Clinical data
☒	☐	Dual use research of concern

Methods

n/a	Involved in the study	
☒	☐	ChIP-seq
☒	☐	Flow cytometry
☒	☐	MRI-based neuroimaging

Antibodies

Antibodies used	Primary antibodies:<br>- rabbit anti-SOX2 Merck 5603<br>- goat anti-SOX9 R&D 3075<br>- anti-nuclear antigen Novus 235-1.<br>Secondary antibodies:<br>- Donkey anti-Rabbit IgG (H+L) Highly Cross-Adsorbed Secondary Antibody, Alexa Fluor™ 647, Thermo 31573<br>- Donkey anti-Goat IgG (H+L) Cross-Adsorbed Secondary Antibody, Alexa Fluor™ 647, Thermo 21447
Validation	Anti-SOX2: multiple citations of use in mouse tissues in the website from the manufacturer e.g. PMID 26315499<br>Anti-SOX9: multiple citations of use in mouse tissues in the website from the manufacturer e.g. PMID 35294885<br>Novus 235-1: multiple citations of use in mouse tissues in the website from the manufacturer e.g. PMID 34912114

Animals and other organisms

Policy information about studies involving animals; ARRIVE guidelines recommended for reporting animal research

Laboratory animals	Time pregnant mice were from the strain C57BL/6JRj and were obtained from Janvier. Embryos were collected at embryonic days 12.5, 13.5, and 15.5. Embryo sex was not determined.<br>Mice were housed with a standard light/dark cycle and availability of food and water ad libitum.
Wild animals	The study did not involve wild animals
Field-collected samples	The study did not involve field-collected samples
Ethics oversight	All experimental procedures were carried out in accordance to the Swedish and European Union guidelines and approved by the institutional ethical committee in Stockholm County (Stockholms Norra Djurförsöksetiska Nämnd) under ethical permit numbers N155/16 and 20785/2020.

Note that full information on the approval of the study protocol must also be provided in the manuscript.

Human research participants

Policy information about studies involving human research participants

Population characteristics	Breast cancer tissue from one patient was obtained from the Department of Clinical Pathology and Cancer Diagnostics at Karolinska University Hospital, Stockholm, Sweden. Age at diagnosis: 88. Tumor subtype: HER2-positive non-luminal. NHG grade: 3. Tumor size: 30mm. Histological subtype: invasive ductal carcinoma. Lymph node methastasis: yes. Provided in Table S3.
Recruitment	Informed consent was obtained from the participating patient.
Ethics oversight	Experimental procedures and protocols were approved by the regional ethics review board (Etikprövningsnämnden) in Stockholm (2016/957-31, amendment 2017/742-32 and 2021-00795), and informed consent was obtained from the participating patient.

Note that full information on the approval of the study protocol must also be provided in the manuscript.
